# Ergonomic risk factors and work-related musculoskeletal disorders in clinical physiotherapy

**DOI:** 10.3389/fpubh.2022.1083609

**Published:** 2022-12-20

**Authors:** L. J. Fan, S. Liu, T. Jin, J. G. Gan, F. Y. Wang, H. T. Wang, T. Lin

**Affiliations:** ^1^School of Computer Science, Sichuan University, Chengdu, China; ^2^Department of Rehabilitation Medicine, Mianyang Central Hospital, Mianyang, China; ^3^School of Arts, Chongqing University, Chongqing, China; ^4^State Key Laboratory of Oral Diseases, National Clinical Research Center for Oral Diseases, West China Hospital of Stomatology, Sichuan University, Chengdu, China; ^5^Department of Rehabilitation Medicine, West China Hospital of Sichuan University, Chengdu, China

**Keywords:** ergonomic assessment, physiotherapist, Kinect, Rapid Entire Body Assessment (REBA), occupational health and safety

## Abstract

**Objectives:**

The purpose of this study was to objectively quantify and evaluate the ergonomic risk of clinical physiotherapy practices and evaluate physiotherapists for work-related musculoskeletal disorders and pain.

**Methods:**

Twenty-nine physiotherapists in the rehabilitation department of a large-scale tertiary hospital were recruited in this study. The sampling period lasted for 2 weeks for each physiotherapist and interval sampling was adopted to avoid duplication of cases. Therapist posture during physiotherapy was captured, tracked and analyzed in real time using structured light sensors with an automated assessment program. The quantification of ergonomic risk was based on REBA (Rapid Entire Body Assessment) and the RPE (perceived physical exertion) scores of the therapists were recorded before and after treatment, respectively.

**Results:**

Two hundred and twenty-four clinical physiotherapy cases were recorded, of which 49.6% were high risk and 33% were very high risk, with none of the cases presenting negligible risk. The positioning (*p* < 0.001) of physiotherapist had a considerable impact on ergonomic risk and pediatric physiotherapy presented a higher risk to physiotherapists than adults (*p* < 0.001). The RPE score of physiotherapist after performing physiotherapy was greater than before physiotherapy and was positively correlated with the REBA distribution.

**Conclusion:**

Our study creates an automatic tool to assess the ergonomic risk of physiotherapy practices and demonstrates unacceptable ergonomic risk in common practices. The high prevalence of musculoskeletal disorders and pains recommends that rehabilitation assistance devices should be optimized and standard ergonomic courses should be included in physiotherapists' training plans.

## 1. Introduction

Work-related musculoskeletal diseases (WMSDs) are prevalent among healthcare workers, especially those who have direct patient contacts, such as surgeons (1), nurses (2) and therapists (3). Musculoskeletal pain is a possible symptom of WMSD, leading to permanent disability if left untreated (4). As typical disease prevention and post-trauma workers, physiotherapists have sufficient knowledge of musculoskeletal injury prevention strategies. However, they are still at high risk of suffering WMSD, caused by therapy characteristics such as repeating the same tasks, working in the same position for long periods, and treating many patients in a single day (5–7). According to a previous online study, physiotherapists' lifetime prevalence of musculoskeletal pain ranged from 55 to 91% (8), which is the primary factor of physiotherapist absences, with severe implications for productivity and economic benefits. Just as Nordin (9) and Obembe (10) reported, physiotherapists might also become patients.

The high prevalence, severity, and reaction of WMSD among physiotherapists were the main subjects in previous studies (11, 12) and these researches investigated the factors of WMSD among physiotherapists primarily relied on questionnaires. The objective ergonomic assessment has been demonstrated to identify underlying musculoskeletal diseases (13) and ergonomic interventions can be effective in preventing occupational trauma (14). Recently, this has been employed extensively in the field of surgery, especially for ergonomic assessment during surgery with high operative demands, such as endoscopic surgery (15), orthopedic (16) and otorhinolaryngology surgery (17). However, there has been very little objective research on the occupational tasks of physiotherapy (18, 19). Exploring potential ergonomic risk factors in clinical physiotherapy can contribute to targeted interventions at an early stage to protect the musculoskeletal health of physiotherapists, enhance career satisfaction, and ultimately benefit patients.

Therefore, the purpose of this study was to quantify and assess the ergonomic risk in clinical physiotherapy from the perspective of motion analysis, based on the Rapid Entire Body Assessment (REBA) scale, with the assistance of structured light sensor-Azure Kinect, and combined with subjective Perceived Physical Exertion (RPE). An ergonomic assessment program customized specifically for physiotherapists was developed and achieved a high accuracy of assessment, incorporated the behavioral characteristics of physiotherapists. Finally, we provide physiotherapists with comprehensive job safety counseling.

## 2. Materials and methods

### 2.1. Participants

This study was conducted in a large tertiary medical center from November 2021 to October 2022 and obtained ethical clearance from the center (S20220347-01). All 29 (male: 18, female: 11) physiotherapists (age: 30 ± 7) in the Neurological Rehabilitation Clinic of this center were recruited to the study at the medical center, and all participants gave their informed consent. The heights and weights of the participants ranged from 168 ± 9 (mean ± SD) cm and 67 ± 14 kg, and all participating physiotherapists should be qualified to practice physiotherapy and have occupational experience within the last 1 year. Physiotherapists with congenital musculoskeletal disorders, tumors, tuberculosis, and other non-occupational causes of musculoskeletal disorders were excluded, along with therapists who were pregnant. To avoid changes in the participants' posture owing to observation, the nature of the study was kept hidden during the ergonomic evaluation.

### 2.2. Assessment criteria

**Figure 1:**
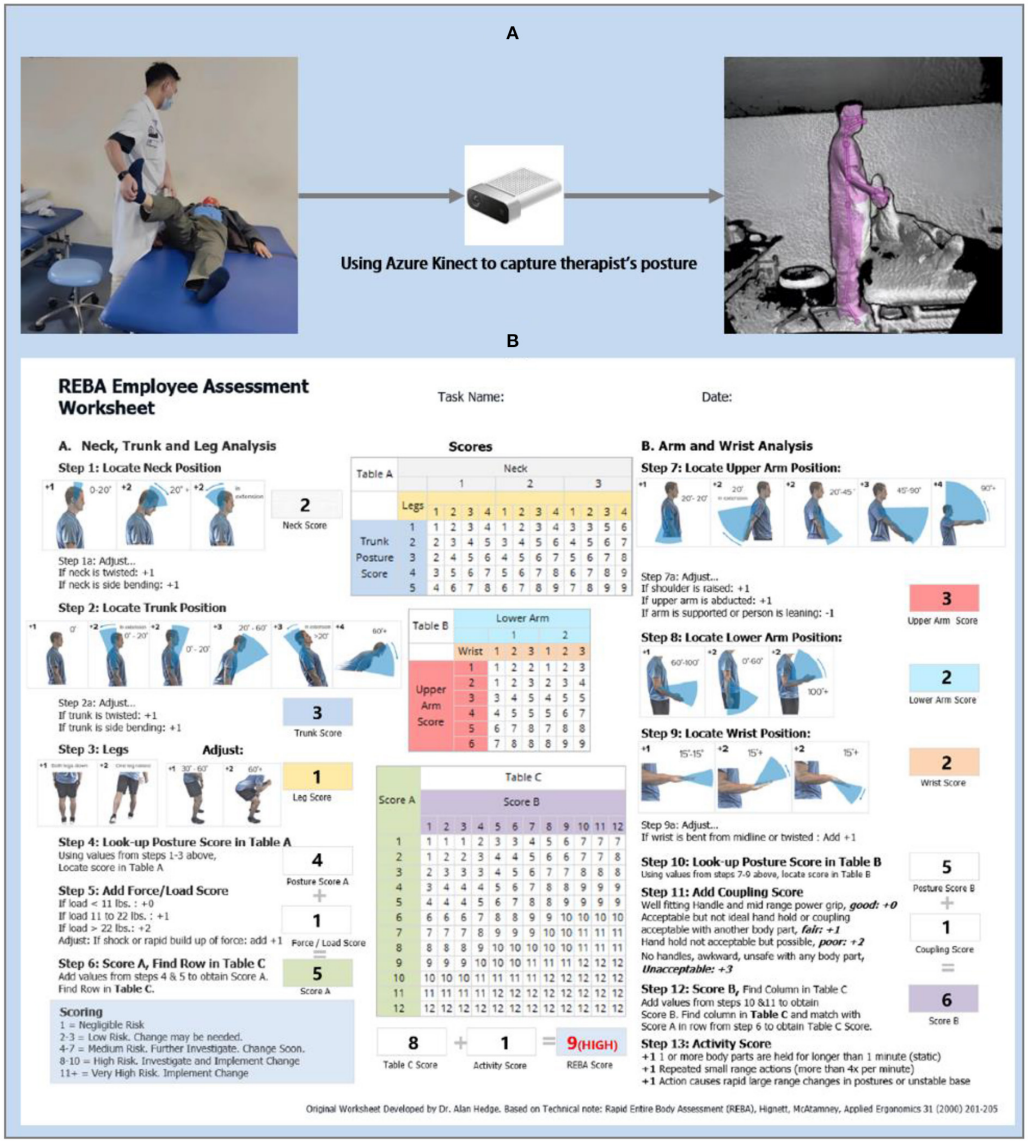
**(A)** Capturing the therapist's posture by Kinect. The leftmost of **(A)** represents the real environment in which physiotherapy is being performed. The middle is the sensor-Azure Kinect, and the rightmost is the modeling of the skeleton of the physiotherapist **(B)**. Example Rapid Entire Body Assessment (REBA) scoring for a physiotherapy.

Rapid Entire Body Assessment (REBA) (20) is an ergonomics risk scoring system that has been demonstrated to be objective and pervasive (Figure 1). It splits the body into separately coded parts (including the trunk, legs, neck, upper arm & lower arm, and twist) and assigns a score to muscle activity in static, dynamic, rapidly changing, or unstable postures. REBA scores range from 0 to 15, with one negligible, 2–3 low risk, 4–7 medium risk, 8–10 high risk, and 11–15 very high risk. Once the REBA score is >11 points, it indicates that an adjustment is required right now.

In this study, REBA was selected as an ergonomic risk assessment standard for several reasons: (1) REBA is convenient to apply and has a favorable cost-effectiveness ratio (21); (2) REBA is determined from the individual scores calculated after assessing each body part, which is consistent with the fact that physiotherapy practice requires the involvement of all parts of the therapists; (3) REBA is also commonly utilized in the medical industry to evaluate the risk of musculoskeletal diseases and ergonomics in health workers (1, 22–25); (4) A group of ergonomists, physiotherapists, occupational therapists, and nurses collaborated to develop REBA. During the development phase, the possible ergonomic risk of physiotherapists was considered entirely, which gave a more accurate assessment of ergonomic risks in physiotherapy.

### 2.3. Data collection

**Figure 2:**
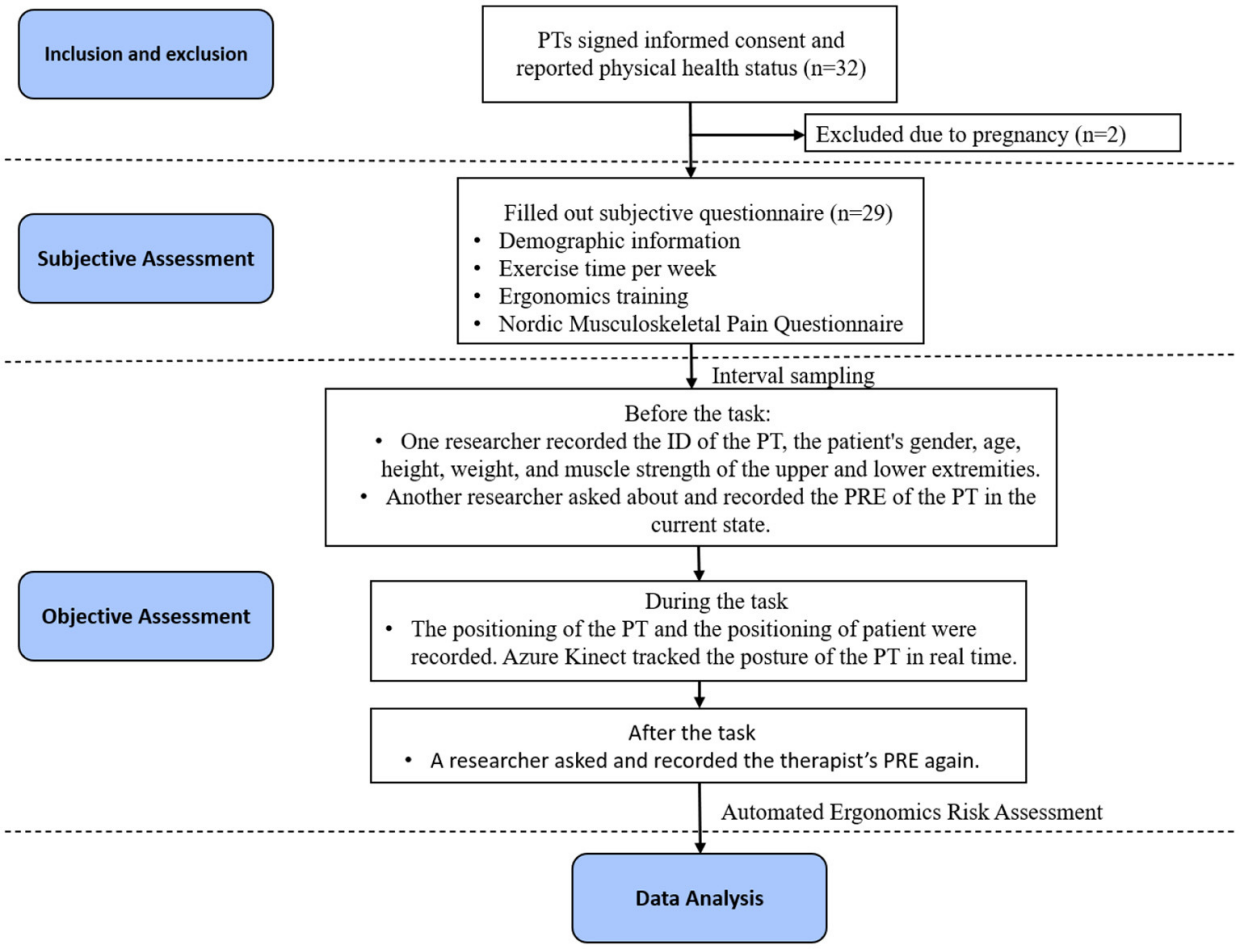
Flowchart: after conducting the inclusion and exclusion of operational cases, 224 cases were included in analysis. Objective assessment was carried out in these cases to collect data from both physiotherapists and patients, followed by a subjective assessment including the demographic information and the body-parts and duration of pain of physiotherapists.

Two researchers who had previously received ergonomics training were responsible for the execution of the experimental protocol, and the study flow is shown in Figure 2. Prior to the start of all observations, the participating physiotherapists were asked to complete a questionnaire that included basic demographic information about age, gender, height and weight [meanwhile, Body Mass Index (BMI) was calculated and recorded from height and weight as a valid indicator of the participants' health status], occupational experience, working hours, work habits, exercise habits, and whether they had received ergonomic training. An adaptation of the Nordic Musculoskeletal Pain Questionnaire (NMPQ) was included in our questionnaire to evaluate painful body parts, period of musculoskeletal pain and pain impact on physiotherapists. Once the questionnaire was completed, a unique ID was generated for each physiotherapist to facilitate subsequent data collection.

To avoid the risk of bias from case duplication, interval sampling was selected, with each physiotherapist sampled for a period >2 weeks. Using the structured light sensor-Azure Kinect to track the therapist's treatment posture during physiotherapy. Azure Kinect is a depth camera for real-time depth sensing and motion capture, which can be used for posture capture and recognition by tracking the skeleton. Compared to earlier Kinect versions, it provides better skeletal tracking accuracy (26). In the past, researchers have experimented with Kinect as one of the automated assessment tools for ergonomics risk (27–29), highlighting its potential for calculating ergonomic risk.

Before each physiotherapy observation, one researcher recorded the PT's ID from the completed questionnaire. Considering the patient was one of the critical factors causing ergonomic risk to physiotherapists, the patient's gender, age, height, weight, and MMST (muscle strength) of the upper and lower extremities were recorded. Another researcher verbally asked and recorded the RPE (Perceived Physical Exertion) of the physiotherapist in the current state. RPE was measured by Borg scale-CR10 (30), which was estimated on a scale of 1 (no stress) to 10 (extreme stress and distress). In addition, a basic calibration of Azure Kinect is required before each observation. Since Azure Kinect has superior skeletal recognition accuracy in non-frontal directional tracking (31), one Azure Kinect was set up on the same side of the physiotherapist with an oblique 45° viewing angle and a fixed distance (2.0 m) and height (1.2 m) to ensure that the physiotherapist's entire body was within the recommended operating range.

During the process of the physiotherapy observation, the positioning of the therapist and the patient was recorder, and one researcher controlled Azure Kinect to capture and track a therapist's posture in real time. We chose a fixed time during the core treatment phase for each case to record data in real-time using our automated tools, which converts joint angle data into REBA score data. The depth image of posture and REBA scores for each body part were all recorded in the data and the overall risk score was a time-weighted average of the percentage of time spent on each REBA score. At the end of the physiotherapy task, one researcher recorded the RPE of the physiotherapist again.

During the sampling phase, 235 physiotherapy cases were recorded in the clinical setting. To avoid the risk of bias, we filtered 11 cases prior to formal analysis due to duplicate sampling of patients, ultimately retaining 224 physiotherapy cases.

### 2.4. Assessment of ergonomics

Traditionally, the ergonomic score is measured by experts' calculation based on the behavior of experiment participants using paper and pen. It is a tedious, time-consuming and inefficient task. Furthermore, as ergonomic score criteria typically consist of subjective and objective judgment, the inconsistency between experts may increase on account of subjective judgment. To address these difficulties, we created the first automatic ergonomic risk assessment tool tailored to the needs of physiotherapists and their practices. Our tool captures the physiotherapist's posture by Azure Kinect and annotates the physiotherapist's skeleton in real-time. If human bones are occluded and blurred in the process of capture, the bone features at this time will automatically match with the bone instances in the standard library, and fill in the missing values with the instances with the highest similarity. After the pose is determined, the transformation's intermediate parameters can be calculated using the linear angle algorithm based on the values of coordinates, rotation, and offset of each bone joint, and length in the bone. Then, the intermediate parameters will be templated with REBA criteria to get the final ergonomic score and risk level.

**Figure 3:**
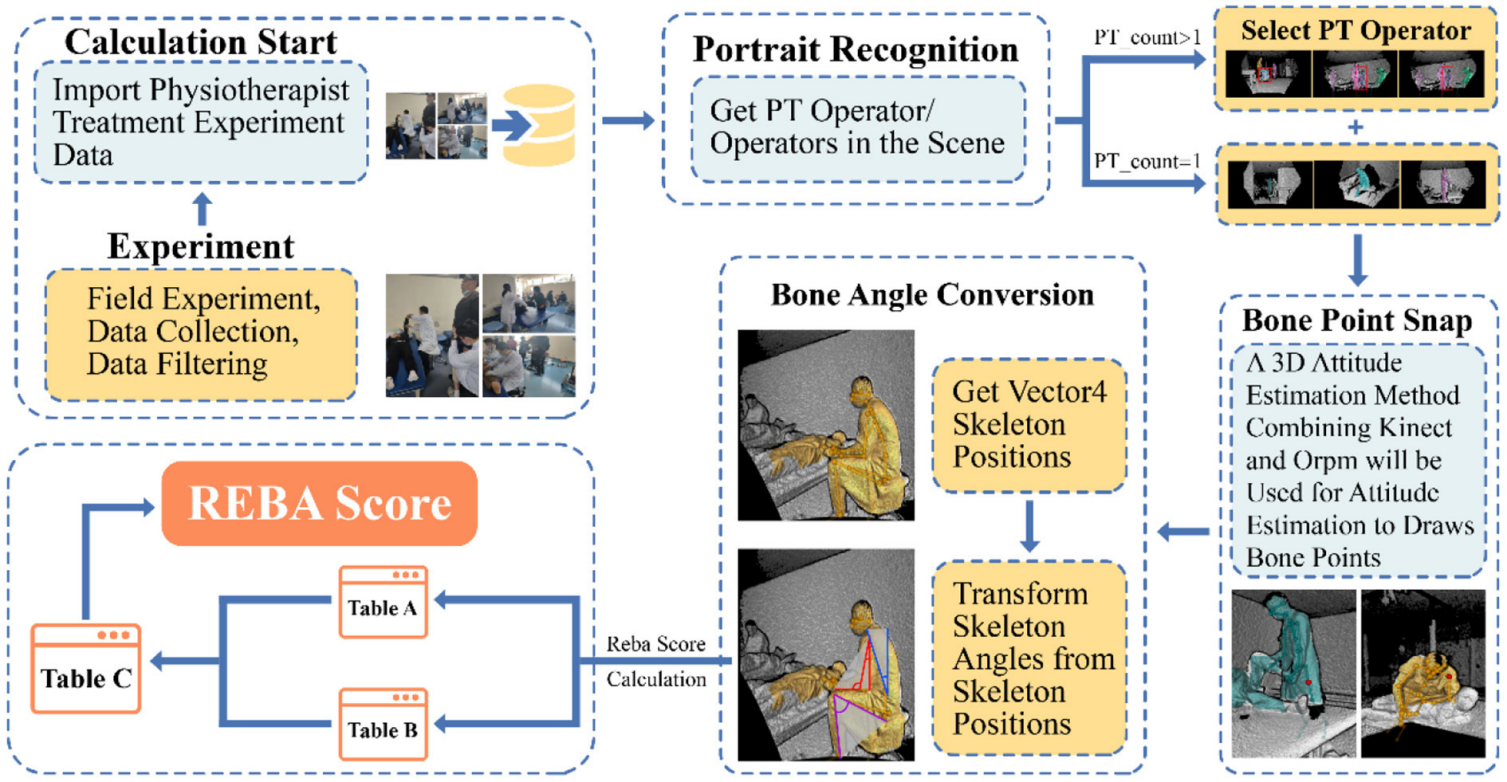
Procedures of automatic ergonomics assessment tools. Once Kinect has scanned the spatial environment and the person, the researcher can select the person index to capture the motion of the specified person within the scene. Confirming the capture object, the automated algorithm will calculate the angle value based on the bone joints and bone length, which finally corresponds to the REBA score “+” (The REBA score is a combination of REBA Table A and REBA Table B). The generated data will be saved to the local port in real time.

The process of automatic assessment is depicted in Figure 3. Firstly, once Kinect scanned the scene, an image edge recognition approach based on the random forest is utilized to capture therapists in the scene. Then, traversing the coordinates of the selected therapist's bone joints from the application programming interface of Kinect body tracking. After traversing the coordinates and rotation of 20 bone joints, the relative angle between relative bone joints of the skeleton is used to calculate REBA score.


$$\begin{array}{l} \text{Join}{\text{t}}_{\text{n}-1}\;=\;\left(x_{n-1},y_{n-1},z_{n-1}\right)\\ \;\text{Join}{\text{t}}_{\text{n}}\;=\;\left(x_{n},y_{n},z_{n}\right)\\ \text{Join}{\text{t}}_{\text{n}+1}=\left(x_{n+1},y_{n+1},z_{n+1}\right) \end{array}$$

Which x, y, and z denote the value of the x-axis, y-axis and z-axis (value of x-axis and y-axis from −1 to 1, the value of z-axis represents the distance between Kinect and the physiotherapist). The offset angle of the upper arm,


$$\begin{array}{l} {\text{θ}}_{\text{UpperArm}}\;=\;\text{arccos}\left(cos\;{\text{θ}}_{\text{UpperArm}}\right)\\ =\;arccos\left(\frac{Vector_{Joint_{n-1}-Joint_{n}}•Vector_{Joint_{n}-Joint_{n+1}}}{\left|Vector_{Joint_{n-1}-Joint_{n}}\right|\;*\;\left|Vector_{Joint_{n}\;-\;Joint_{n\;+\;1}}\right|}\right)\\ Vector_{Joint_{n\;-\;1}\;-\;Joint_{n}}\;=\;\left(x_{n-1}-x_{n},\;y_{n-1}-y_{n},z_{n-1}-z_{n}\right)\\ Vector_{Joint_{n}\;-\;Joint_{n\;+\;1}}\;=\;\left(x_{n}-x_{n+1},y_{n}-y_{n+1},z_{n}-z_{n+1}\right) \end{array}$$

After getting the offset angle of the left upper arm, it was compared to the template of REBA. The score was composed of a basic score and an additional score. For the basic score of the upper arm, two points for a forward extension angle of 20°-45°, 3 for 45°-90°, and 4 for more than 90°. In addition, if the arm was abducted or rotated and the shoulder was raised, judged by the rotation value, both added 1 point to the basic score. At last, a non-linear accumulation of each part's score was performed to assess the ergonomic risk of the physiotherapist.

It was worth noting that physiotherapy was frequently practiced in a setting where multiple physiotherapists were operating on different patients at the same time, with obscurity between physiotherapists and physiotherapists, as well as between physiotherapists and the environment, resulting in inaccuracy in the process of physiotherapist posture capture. To address this issue, first we provided a more pristine experimental site and avoided incomplete posture capture by standardizing the position parameters of Azure Kinect to the extent. Then, the typical physiotherapy posture was employed as a compensatory element to improve the capture, assessment accuracy, and robustness. When the captured joint angle of the physiotherapist is greater than the normal physiological bending threshold, it is considered as an abnormal masking of that joint angle. Once abnormal tortuosity occurs, missing values are assigned to the collected skeletal information. At this point, the missing values are filled by traversing the pose examples with the highest similarity by comparing the skeletal angles that do not contain abnormal occlusion site with the skeletal angles of each posture in the pose library. The examples in the Physiotherapy Standard Posture Library are derived from typical physiotherapy tasks, which are drawn from physiotherapy literature, research (32, 33), interviews and observations of physiotherapists.

To evaluate the accuracy of the automatic tool in measuring REBA score, we randomly sampled 15 static postures from 224 physiotherapy cases as pre-test data. The REBA scores of these 15 postures were assessed by three experts (a doctor with 5 years of experience in occupational disease rehabilitation and two researchers in the field of ergonomics), and the means were used as the baseline. Compared to the experts' assessment, the accuracy of the measurements was 80% for the trunk, 92.3% for the neck, 86.7% for the legs, 86.7% for the upper arms, 100% for the lower arms, and 86.7% for the wrists. The discrepancy between expert and automatic tool scores was no more than two points for each part. The trunk was measured with 80% accuracy, the neck 92.3%, the legs 86.7%, the upper arms 86.7%, the lower arms 100% and the wrists 86.7%. For each part, the discrepancy between expert and automatic tool scores was no more than two points.

### 2.5. Statistical analysis

We used expert assessments as a baseline to evaluate the tool's accuracy. We initially utilized univariate regressions to discover which physiotherapy variables were linked to higher REBA scores. In a multivariate model, a value of *P* < 0.1 was included. Wilcoxon paired rank sum test were performed for differences in RPE score before and after physiotherapist manipulation and the Spearman test was used to assess the correlation between PRE difference and REBA scores. Initial analysis of musculoskeletal pain in the last week was performed using logistic regression, and a multivariate model was assembled using the same approach as for REBA score analysis. As potential factors, survey factors and the median REBA score were employed. The coefficients for each variable, as well as their corresponding *P* values, are presented.

## 3. Results

### 3.1. Ergonomic evaluation results

Two hundred and twenty-four clinical physiotherapy practices of 29 therapists were recorded, with assessments ranging from 5 to 15. All of the 29 participants responded to the questionnaire and the demographic information of the participants is presented in Table 1. Of those 224 observations, 132 were adult physiotherapy sessions and 92 were pediatric physiotherapy sessions. The physiotherapists were observed 127 in a sitting position, 68 in a standing position, and 29 in a kneeling position. No physiotherapy case was found to be a negligible and low ergonomic risk, with 82.59% of physiotherapy practices being high or very high (Table 2). During the study, one physiotherapist worked with only one patient at a time, and all of them provided non-device-assisted therapy, primarily manual therapy. As a result, the use of rehabilitation assistance devices and the presence of other therapists were excluded. In addition, physiotherapy for the same patient was excluded to reduce the risk of bias.

**Table 1:** Demographics of participating physiotherapists.

**Demographic**	***n* = 29**
PT age (M ± SD)	30 ± 7
PT height (cm)	168 ± 9
PT weight (kg)	65 ± 14
PT BMI	23 ± 3
PT OE (years)	7 ± 6
PT gender (n/%)	
Male	18(62%)
Female	11(38%)
Ergonomic education	
Yes	6(21%)
No	23(79%)

PTs, Physiotherapist; BMI, Body Mass Index; “OE” is a short forms of “Occupational Experience,” “n” represents the number of participating physiotherapists”.

**Table 2:** Physiotherapy stratified by ergonomic risk level as determined by REBA.

**Percent of PTs by REBA risk levels**					
	**Risk level**				
**Operation object**	**Negligible**	**Low**	**Medium**	**High**	**Very high**
Adult (*n* = 132)	0.0	0.0	25.76	50.76	23.48
Child (*n* = 92)	0.0	0.0	5.43	47.83	46.74
Total (*N* = 224)	0.0	0.0	17.41	49.55	33.04

PTs, physiotherapists and the risk level refers to the different levels of ergonomic risk from negligible to very high corresponding to the different thresholds of the REBA score.

**Figure 4:**
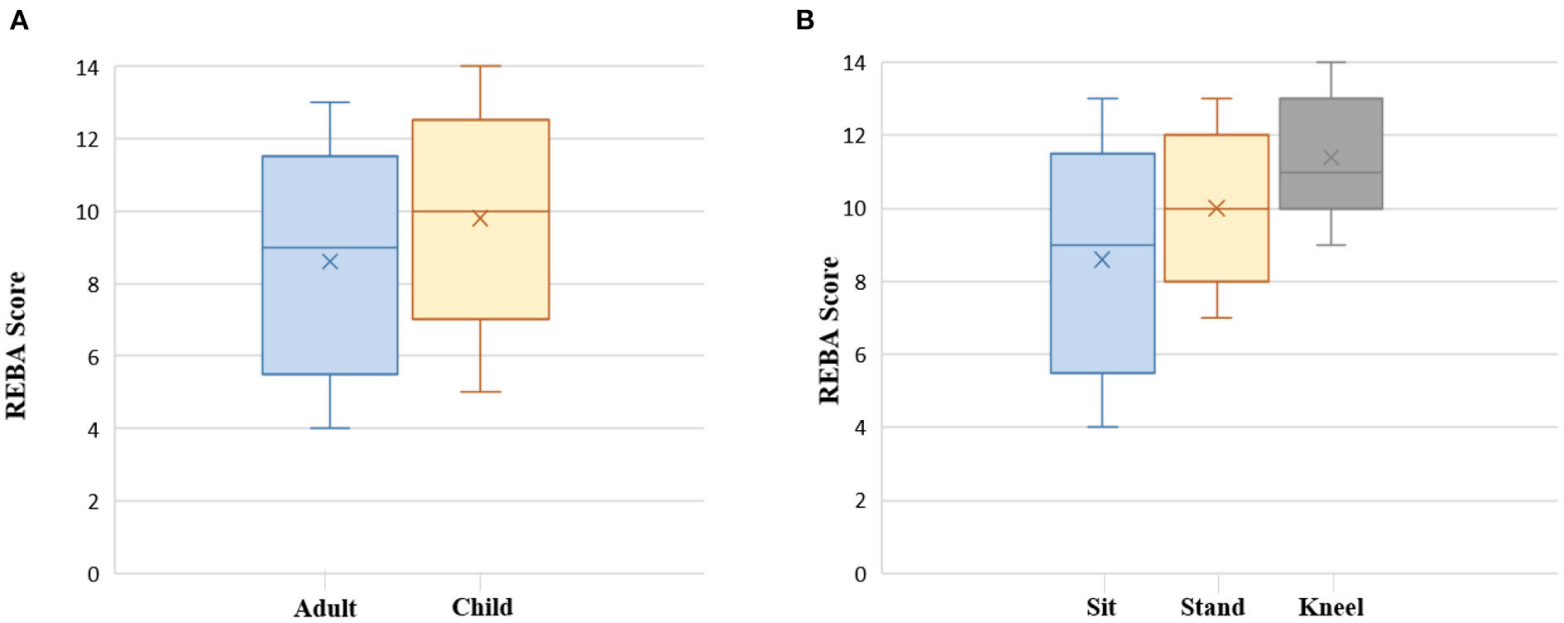
Boxplot displaying the REBA score by physiotherapy on child or adult **(A)**, Physiotherapists positioning **(B)**. The Y-axis is the REBA score and X-axis is the types of parameters .

According to univariate regression, physiotherapists' positioning affected REBA scores. Standing (coefficient = 1.483, *P* < 0.001) and kneeling (coefficient = 2.789, *P* < 0.001) gave the therapist a more ergonomic risk score than a therapist who performed physiotherapy sitting down. When a physiotherapist operated on children, their REBA score was higher than operating on adults (coefficient = 1.263, *P* = 0.001). These physiotherapists' gender, height, and weight were not associated with the REBA risks score, and patients' gender, positioning, and MMST were insignificant (Table 3). A boxplot with significant effects is shown in Figure 4, which is included in multivariate regression. Multivariate regression demonstrated the positioning of standing (coefficient = 1.671, *P* < 0.001) and kneeling (coefficient = 2.479, *P* < 0.001) than seating, and operating on a child (coefficient = 1.173, *P* < 0.001) than an adult predicted higher REBA scores (Table 3).

**Table 3:** Coefficient estimates using each physiotherapy variable to predict REBA scores—Univariate.

**Variable**	**Coef**.	**SE**	***P***
**Univariate regression-REBA**			
PT height	0.015	0.015	0.319
PT weight	0.014	0.009	0.117
PT BMI	0.059	0.036	0.104
PT OE	−0.013	0.024	0.575
**PT gender**			
Male	Ref		
Female	−0.185	0.272	0.561
**PT position**			
Sit	Ref		
Stand	1.483	0.251	<0.001
Kneel	2.789	0.344	<0.001
**Patient type**			
Adult	Ref		
Child	1.263	0.251	0.001
**Patient gender**			
Male	Ref		
Female	0.130	0.265	0.624
**Patient position**			
Sit	Ref		
Lie	0.261	0.455	0.567
Stand	0.150	0.514	0.771
Kneel	−0.190	2.003	0.924
**MMST of arms (patient)**			
≤ 3	Ref		
>3	0.075	0.261	0.774
**MMST of legs (patient)**			
≤ 3	Ref		
>3	0.112	0.262	0.670
**Multivariate regression-REBA**			
**PT position**			
Sit	Ref		
Stand	1.671	0.240	<0.001
Kneel	2.479	0.330	<0.001
**Patient type**			
Adult	Ref		
Child	1.173	0.222	<0.001

PTs, Physiotherapist; OE, Occupational Experience; Coef., Coefficient; SE, standard error. “p” represents the level of significance, and “MMST” is an abbreviation of “Muscle strength,” which can be divided into 5 levels and Level 0 indicates the worst muscle strength, where the patient cannot move and has no movement of the limbs; level 5 indicates normal muscle strength.

Due to the extreme disparities in demographic information between adults and children, a direct investigation of patients' characteristics such as age, height, and weight is an imprudent strategy in all cases. As a result, the child and adult groups were in a secondary analysis on potential factors by the same approach as the primary analysis. However, subgroup analyses of adults and children showed that patient age, height, and weight were not associated with the ergonomic risk to the therapist (Table 4). Similarly, considering therapist height was not significant in the full group analysis. However, the difference in the height of therapists who dealt with patients in a lying position could affect therapist discomfort, so we performed a secondary analysis in the case of patients treated in a lying position (Table 4). Among them, univariate analysis showed that there was still no significant relationship between height of physiotherapists and REBA scores in lying patients.

**Table 4:** Coefficient estimates using each physiotherapy variable to predict REBA scores in children and adults.

	**Coef**.	**SE**	***P***
**Univariate regression—child**			
Patient weight	0.016	0.018	0.371
Patient height	0.005	0.009	0.530
Patient age	0.016	0.058	0.778
**Univariate regression—adult**			
Patient weight	−0.001	0.017	0.956
Patient height	0.010	0.025	0.675
Patient age	0.021	0.614	0.140
**Univariate regression—lying position**			
PT height	0.027	0.014	0.124

PTs, Physiotherapist; Coef., Coefficient; SE, Standard Error. “p” represents the level of significance. Regression analyses of patient demographic information (age, height, weight) were performed separately in the subgroups of children and adults. No multifactorial analysis was involved as the univariate regression showed no variables fulfilling p <0.1.

### 3.2. Results of the RPE

There was a significant difference in the RPE reported by the physiotherapists before and after the execution of the physiotherapy and the RPE score was higher after the treatment compared to the pre-treatment [median 2 (1–3) and 6 (2–8) respectively; *P* < 0·001]. Spearman's correlation coefficient suggested that the difference in RPE showed a positive correlation with REBA scores (coefficient = 0.697, *P* < 0.001).

### 3.3. Results of the questionnaire

All physiotherapists involved in this study participated in the subjective survey. The results revealed that the physiotherapists had a significant prevalence of musculoskeletal discomfort, particularly in the neck, shoulders, and lower back. 79.3% of respondents reported musculoskeletal discomfort (pain or numbness) in at least one body part appearing in the last year through NMPQ, with neck discomfort being the most common (91.3%), followed by lower back (56.5%), shoulders (52.2%), knees (47.8%), hips/thighs (43.5%), hands/wrists/fingers (39.1%) and upper back (34.8%). 62.1% of participants reported that musculoskeletal discomfort prevented them from performing normal activities, and 24.1% went to see a doctor or physician for this condition.

Univariate regression demonstrated that weekly activity and BMI of therapist (coefficient = 0.356, *P* = 0.038) were linked to whether the therapist had musculoskeletal discomfort. Physiotherapists who exercised > 4 h per week had a decreased risk of pain than those who exercised <4 h per week (coefficient = 1.872, *P* = 0.025). As the median REBA score rises, the likelihood of physiotherapists suffering discomfort rises (coefficient = 2.823, *P* = 0.003). The physiotherapist's height, weight, gender, occupational experience, frequency of utilizing rehabilitation assistive devices, and level of ergonomic training were not significant factors of musculoskeletal discomfort (Table 5).

**Table 5:** Coefficient estimates obtained from univariate and multivariate logistic regression predicting musculoskeletal discomfort.

	**Coef**.	**SE**	***P***
**Univariate regression**			
Sex			
Male	Ref		
Female	0.270	0.775	0.728
Age	0.034	0.056	0.549
Occupational experience	0.029	0.064	0.646
PT height	−0.004	0.043	0.933
PT weight	0.051	0.032	0.112
PT BMI	0.356	0.171	0.038
Exercise hours/week			
≤ 4	Ref		
>4	1.872	0.838	0.025
Use of assistive devices			
Never	Ref		
Sometimes	0.288	1.155	0.803
Often	−0.272	1.049	0.796
Ergonomics education			
No	Ref		
Yes	−0.431	0.963	0.655
REAB score	2.823	0.954	0.003
**Multivariate regression**			
Exercise hours/week			
≤ 4	Ref		
>4	−3.354	1.303	0.185
PT BMI	0.552	0.355	0.120
REAB score	2.857	1.248	0.022

PTs, Physiotherapist; Coef., Coefficient; SE, standard error; “p” represents the level of significance. The REBA score here refers to the median score of REBA of multiple treatments collected by each physiotherapist.

## 4. Discussion

The purpose of this study was to objectively investigate the ergonomic risks and potential high-risk influences in clinical physiotherapy. We then combined structured light sensing technology with computer science techniques to automate motion capture, motion tracking and motion analysis of the therapist during physiotherapy. This study builds upon some works which have confirmed the high prevalence of musculoskeletal disorders and pains in the department of rehabilitation medicine, particularly among physiotherapists (34–36). However, most previous studies relied on questionnaires, and there are very few quantitative studies on physiotherapists' ergonomic risks.

EMG (Electromyogram) and motion analysis are two main methodological tools used for quantifying physical workload and muscular discomfort, both of which can identify abnormal muscle activity patterns related to abnormal kinematic patterns (37). Early studies have shown that fatiguing contractions of a muscle were consistent with typical changes during the EMG time course (38, 39). Moreover, the measurement of sEMG (surface EMG) in occupational settings is preferable because of its non-invasive recording and a minimal restraint of subjects (40). Riggle (41) previously studied muscle loading in a group of surgeons by sEMG to distinguish differences in ergonomic risk caused by different surgical instruments. In addition, Armijo (15) also used surface EMG sensors to explore differences in muscle loading among surgeons of different genders. For physiotherapists, Yoopat aimed to assess the different workloads of physiotherapists within three groups of varying work experience (18), the maximum voluntary contraction of trapezius and deltoid muscles were tested using electromyography. As biomechanical factors (posture, force, and duration of tasks) are the most significant factors related to the workstation (42), researchers have developed a series of models based on these factors to assess the ergonomics risk of given tasks, such as rapid entire body assessment (REBA) (20), rapid upper limb assessment (RULA) (43) and Postural Loading on the Upper Body Assessment (LUBA) (44). Researchers can utilize optical-based sensors and wearable-based inertial measurement units (IMUs) for motion capture and analysis to obtain more accurate ergonomic risk results.

However, the wearable EMG and IMUs create discomfort for the therapist. To circumvent this problem, we used Azure Kinect, a sensor based on structured light technology. Azure Kinect is capable of capturing and tracking human skeletal motion for motion analysis based on computer vision technology and are often deployed in objective assessments of ergonomic risk. To investigate the difference in ergonomic load between standing and sitting during simulated endoscopic sinus surgery, Lobo used the Kinect sensor to record six surgeons performing simulated procedures on five cadaveric heads, demonstrating a higher ergonomic load in the standing position (45). A recent study assessed the ergonomic risks of six typical physiotherapy postures using Kinect. The study recruited some students to simulate these postures and the whole experiment was conducted in a laboratory environment. As a pilot study, it illustrates to some extent the non-negligible ergonomic risks involved in physiotherapy tasks. However, the selected postures, fixed laboratory environment, and conducted with a simulated patient may not reflect the real situation in physiotherapy procedures and potential ergonomic risks (19). Besides posture, electromyography is also widely used in studies related to musculoskeletal discomfort and workload (15, 41), sometimes combined with posture (46).

This cross-sectional research focuses on assessing the ergonomic risks in physiotherapy Procedures and possible predictors of musculoskeletal discomfort and pain. We aimed to create an innovative and novel ergonomic risk assessment tool exclusively for physiotherapy operations. Our technology has a consistent accuracy rate compared to typical expert measurements and can minimize assessment time by over 95%, considerably boosting assessment efficiency. Unlike previous automatic ergonomic assessment programs, this tool is more focused. When incomplete angle capturing occurs due to obscuration, this tool will select the skeleton in the standard pose library with the greatest similarity to complete the uncaptured skeletal joints. The poses in the standard pose library were derived from typical physical therapy tasks derived from the physical therapy literature, observations of physical therapists, and interviews. The researchers confirmed the availability of the library by communicating with senior clinical rehabilitation doctors and physiotherapists, and developed an automatic evaluation tool based on Kinect. Kinect is a depth image acquisition sensor based on far-light infrared sensing that can avoid privacy concerns for patients and clinicians at the acquisition site. Data acquisition can be made without disturbing the physician or causing further interruption compared to standard wearable sensing—Inertial Sensing Unit (IMU).

Observations and assessment of 224 physiotherapy cases revealed that all physiotherapy practices have a non-negligible ergonomic risk, with a high risk of 49.55% and a very high risk of 33.04%. Our study demonstrated that when a physiotherapist provided therapy to children, the physiotherapist was exposed to more severe ergonomic risks than when dealing with adults. It is because children's size is smaller than adults, and the physiotherapist must provide additional protection for the child during physiotherapy to avoid injury. Secondly, children are reluctant to cooperate with physiotherapists, and more efforts needed to be made by the physiotherapist to fix the child in a therapeutic position. For this reason, we advocate a rational allocation of physiotherapists' rest time and task performance from a health economics perspective, which contributes to muscle recovery occurrence (47) and increased occupational satisfaction. Additionally, we discovered that kneeling postures pose a higher risk for therapists than sitting or standing during physiotherapy, thus some new ergonomic chairs should be utilized to account for this poor body positioning. We hypothesized that differences in MMST of patients might act as one predictor of changes in ergonomic risks, while no significant association was found after univariate regression analysis. We believe this was because this research focused on direct contact physiotherapy procedures like manual therapy, where the patient, primarily as a passive recipient, was in relaxation and did not require the assistance of muscle strength. In this study, patients' posture was not a significant predictor associated with ergonomic risk. After grouping cases with adults and children, univariate regression analysis showed that age, height and weight were insignificant to ergonomic risk.

The subjective survey indicated that physiotherapists have a high incidence of musculoskeletal discomfort, with 92.3% suffering from pain, discomfort, or numbness in at least one region in the previous year, with neck discomfort being the most common. The exercise hours per week and the median REBA score for physiotherapists significantly correlate with musculoskeletal discomfort in the last 7 days. Physiotherapists with a higher REBA are more likely to have had musculoskeletal discomfort and pain in the previous 7 days, consistent with earlier research (48). Furthermore, physiotherapists who exercised for fewer than 4 h per week were more likely to experience musculoskeletal discomfort than those who exercised for more than 4 h. The gender, age, height, the weight of the physiotherapist and frequency of assistive device use had no significant effect on musculoskeletal discomfort in subjective study. Future research should increase the sample size to determine these relationships conclusively. Besides, this study has confirmed the differences in ergonomic risk related to the positioning of physiotherapists and practicing on adults or children. It is essential to concentrate on the differences caused by the patient's type of illness (e.g., hemiplegia, fracture, or discomfort) and type of healthcare (e.g., Physiotherapy in a normal PT room or the intensive care unit) in subsequent research, this would help to advance positive health economics in physiotherapy and provide physiotherapists with more affordable benefits.

An ergonomic intervention aimed at improving physiotherapy posture and reducing musculoskeletal discomfort is warranted, which is closely related economy and productivity (49). Physiotherapy education should focus not only on the ergonomics of the patient but also on the ergonomics of the physiotherapist. Only 5 physiotherapists polled had received formal ergonomics training. Re-standardizing physiotherapy postures and including ergonomic risk prevention courses in physiotherapy education are excellent ways to reduce musculoskeletal discomfort. At the end of the ergonomic evaluation, we randomly interviewed several physiotherapists, and some of them indicated that in addition to a lack of ergonomic education, physiotherapy facilities and rehabilitation assistance devices were a hindrance to our work sometimes.

Our study conducted an objective ergonomic risk assessment of physiotherapists in a clinical physiotherapy scenario and came up with some novel findings. However, there are some limitations to our study. First, the diagnosis of WMSDs using self-reported cases inevitably led to recall bias, and more specific clinical diagnostic criteria are needed in the clinical diagnosis and treatment of the disease. Then, although 224 cases were recorded, we used the patient's muscle strength level instead of the patient's disease type, and the diversity of cases was not considered complete. Future work should increase the sample size to establish these relationships conclusively.

## 5. Conclusion

In this study, we developed the first automatic ergonomic assessment tool specially designed for physiotherapists. Potential risk factors known to be associated with musculoskeletal discomfort were identified in our study. We also demonstrated a high prevalence of musculoskeletal discomfort in the physiotherapy cohort, and a lack of ergonomic training. Future research must reduce ergonomic risk by optimizing physiotherapy assistance devices and re-standardizing operating positions.

## Data availability statement

The raw data supporting the conclusions of this article will be made available by the authors, without undue reservation.

## Ethics statement

The studies involving human participants were reviewed and approved by Mianyang Central Hospital Ethics Review Committee. The patients/participants provided their written informed consent to participate in this study. Written informed consent was obtained from the individual(s) for the publication of any potentially identifiable images or data included in this article.

## Author contributions

All authors together conceived and developed the study under the lead of LF and SL. TJ, FW, HW, JG, and TL developed the questions, survey methods and participated in the analysis and writing. LF led the first draft with SL and the entire team discussed results and contributed to the final manuscript. All authors read and approved the final draft of the manuscript.
